# A systematic review of studies investigating the acute effects of *N*-methyl-*D*-aspartate receptor antagonists on behavioural despair in normal animals suggests poor predictive validity

**DOI:** 10.1177/23982128221081645

**Published:** 2022-03-12

**Authors:** Martin Viktorov, Matthew P. Wilkinson, Victoria C. E. Elston, Medi Stone, Emma S. J. Robinson

**Affiliations:** School of Physiology, Pharmacology & Neuroscience, University of Bristol, Bristol, UK

**Keywords:** Rapid-acting antidepressant, *N*-methyl-*D*-aspartate, behavioural despair, predictive validity, mice, forced swim test, tail suspension test

## Abstract

The ability of the *N*-methyl-*D*-aspartate receptor antagonist ketamine to induce a rapid and sustained antidepressant effect has led to a surge in pre-clinical studies investigating underlying mechanisms and seeking novel treatments. Animal models are key to this research as they can provide a behavioural readout linking underlying mechanisms to clinical benefits. However, quantifying depression-related behaviours in rodents represents a major challenge with the validity of traditional methods such as models of behavioural despair (forced swim test and tail suspension test) a topic of debate. While there is good evidence to support the value of using these behavioural readouts to study the effects of stress, these approaches have largely failed to detect reliable phenotypic effects in other disease models. In this systematic review, we identified publications which had tested *N*-methyl-*D*-aspartate receptor antagonists in normal animals using either the forced swim test or tail suspension test. We compared findings for different doses and time points and also drugs with different clinical profiles to investigate how well the outcomes in the rodent model predicted their effects in the clinic. Despite clear evidence that *N*-methyl-*D*-aspartate receptor antagonists reduce immobility time and hence exhibit an antidepressant profile in these tasks, we found similar effects with both clinically effective drugs as well as those which have failed to show efficacy in clinical trials. These findings suggest that behavioural despair tests in normal animals do not provide a good method to predict clinical efficacy of *N*-methyl-*D*-aspartate receptor antagonists.

## Introduction

Major depressive disorder (MDD) is a debilitating mental health condition with major social, economic and personal consequences that is increasing in prevalence in modern society (World Health Organisation, 2020). Its core symptoms include persistent feelings of hopelessness, loss of interest in previously rewarding activities, fatigue and impaired motivation (*Diagnostic and Statistical Manual of Mental Disorders* (5th ed.; DSM-V)). MDD is the leading cause of disability adjusted life years worldwide and an estimated 800,000 people commit suicide each year (World Health Organisation, 2014). The current first-line antidepressants target monoamine systems with the most prescribed drugs being the selective serotonin re-uptake inhibitors (SSRIs, NICE, 2016). However, their delayed therapeutic onset and lack of efficacy in an estimated 20% of patients highlights the need for treatments with more reliable therapeutic outcomes (Serafini et al., 2014)

Ketamine is a high affinity *N*-methyl-*D*-aspartate receptor (NMDAR) antagonist with affinity at several secondary targets including µ-opioid receptors, Σ-receptors, nicotinic acetylcholine receptors and L-type Ca^2+^ channels (Duman, 2018; Zorumski et al., 2016). Ketamine has been used historically as an analgesic and anaesthetic but in 2000, low dose ketamine was shown to induce an acute antidepressant effect which was sustained long after its initial pharmacological actions (Berman et al., 2000). Low dose ketamine can reduce suicide ideation in treatment-resistant patients within 24 h of administration (Wilkinson et al., 2018) and nasal S-ketamine has recently been licensed for use in treatment-resistant MDD (Mahase, 2019). Evidence that the metabolite of ketamine, hydroxynorketamine (HNK) induces similar antidepressant effects but linked to actions at α-amino-3-hydroxy-5-methyl-4-isoxazolepropionic acid (AMPA) rather than *N*-methyl-*D*-aspartate (NMDA) receptors (Zanos et al., 2016) and a lack of consistent clinical findings with other NMDAR antagonists (Heresco-Levy et al., 2006, 2013; Kishi et al., 2017; Preskorn et al., 2015, 2008; Sanacora et al., 2014, 2017) has led to questions about whether the NMDAR is the primary target mediating the rapid-acting antidepressant (RAAD) effects of ketamine. One of the most widely used methods to investigate RAADs in rodent models is to quantify the effects of drug treatments using either the forced swim test (FST) or tail suspension test (TST) which were developed as assays to predict antidepressant efficacy (Slattery and Cryan, 2014). These methods are simple and provide fast results which are largely replicated across different laboratories making it a popular behavioural readout linked to antidepressant effects (Krishnan and Nestler, 2011). While the original FST was validated as a test to predict antidepressant drugs acting through monoaminergic targets (Porsolt et al., 1977), its use has expanded to other areas including disease models and non-monoamine pharmacology. Both tests subject rodents to stress by creating inescapable conditions and then measure time taken for rodents to cease attempting to escape (see Slattery and Cryan, 2012 for detailed protocol). The drug-induced reduction in immobility (used as a measure of behavioural despair) is seen when conventional antidepressants are tested in these assays are predictive of antidepressant effects (Slattery and Cryan, 2014; Yankelevitch-Yahav et al., 2015). Although with good predictive validity for monoaminergic antidepressants, these models of behavioural despair are not thought to be valid when looking at depression-related phenotypes or when characterising non-monoaminergic drugs (Slattery and Cryan, 2014). It is now thought that the FST and TST measure the ability to cope with stress (Commons et al., 2017) alongside the speed that animals change from an acting to a passive coping strategy (Molendijk and de Kloet, 2015). However, the lack of evidence for impairments in other putative models of depression such as early life adversity (Dimatelis et al., 2016; Lee et al., 2014) does not support their use in the study of disease models. Behaviours in the FST and TST are also affected by factors unrelated to depression including locomotor activity (Can et al., 2012; Commons et al., 2017; Porsolt et al., 1977; Reardon, 2019; Slattery and Cryan, 2014; Yankelevitch-Yahav et al., 2015). This is especially problematic when considering drugs like ketamine which has been observed to increase rodent locomotion at doses as low as 4 mg/kg (Imre et al., 2006).

The aim of this systematic review was to investigate whether the data for different NMDAR antagonists tested in the FST and TST in normal mice suggested the assay was predictive of their clinical efficacy. We initially selected all publications which had used an NMDAR antagonist in the FST and TST in mice and then excluded studies which had included a disease model. These limits were used to restrict the total number of publications for more in-depth analysis but do mean the findings may not be relevant to studies in other rodents or in disease models. A systematic review by Polis et al. (2019) has previously reported reduced immobility in mice following ketamine administration. There were, however, mixed results depending on whether the test involved stressed or non-stressed animals but did not expand to other NMDAR antagonists (Polis et al., 2019). Alongside the main measure of immobility time, we also collected data relating to locomotor activity, pharmacokinetics and indices of study quality (Hooijmans et al., 2014). Not all drugs included in this systematic review have been tested in patients but where data are available, we discuss how well findings in the FST/TST predict clinical efficacy.

## Methods

### Search strategy

We conducted a comprehensive systematic review and meta-analysis protocol in accordance with PRISMA guidelines (Moher et al., 2009). A search strategy was implemented in PubMed containing the search terms ‘ketamine’ or ‘NMDA antagonist’ and ‘forced swim test’ or ‘tail suspension test’ with all searches including the term ‘mice’. The reference list of included studies was also assessed for relevant publications that were added to the initial pool of studies. Studies were not limited by language or year of publication.

### Study selection

**Figure 1.**
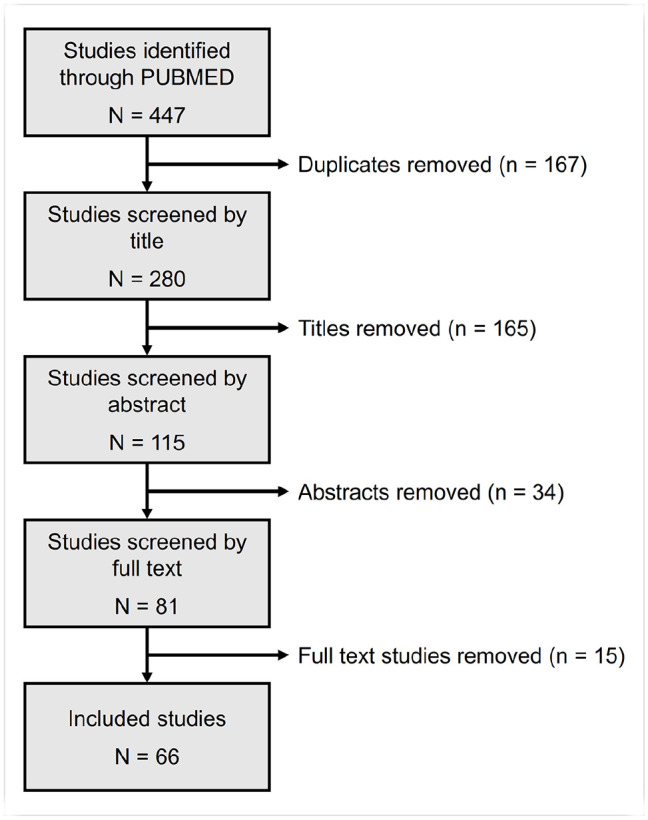
Flowchart of study selection process for meta-analysis.

Following removal of duplicate studies, records were screened by title (see Figure 1) according to the pre-defined inclusion criteria. Studies were required to be primary literature containing wild-type mice that were acutely treated with an NMDAR antagonist in either the FST or TST. Studies where additional manipulations were used, such as chronic social defeat stress or administration of other pharmacological compounds, prior to NMDAR antagonist administration were excluded. Following title screening, records were next reviewed by their abstract and then by full text which resulted in a final data set of 66 studies for analysis. All screenings were conducted by two independent reviewers (M.S. and V.C.E.E.) with discrepancies mediated by a third reviewer (E.S.J.R.). Prior to publication an additional search with the same criteria was conducted by M.V. and M.P.W. to identify relevant studies published during this review process. Although not an NMDAR antagonist, the metabolite of ketamine, HNK, was also included in the analysis where papers investigating it met the inclusion criteria. A summary of the different NMDAR antagonists included in the analysis is given in Table 1.

**Table 1.** Summary of included NMDAR antagonists.

Drug name	Mechanism of action	Number of replicates^a^	Data available^b^			
			⩽60 min	24 h	>24 h	FST/TST
Ketamine	Non-competitive NMDAR antagonist	116	Y	Y	Y	Both
(R)-Ketamine	Non-competitive NMDAR antagonist	12	Y	Y	Y	Both
(S)-Ketamine	Non-competitive NMDAR antagonist	13	Y	Y	Y	Both
4-Cl-KYN	Non-competitive NMDAR antagonist	11	Y	Y	N	Both
MgSO_4_	Non-competitive NMDAR antagonist	4	Y	N	N	Both
Memantine	Non-competitive NMDAR antagonist	8	Y	N	N	TST
Methoxetamine	Non-competitive NMDAR antagonist	6	N	Y	N	Both
MK-801	Non-competitive NMDAR antagonist	65	Y	N	N	Both
(+)-MK-801	Non-competitive NMDAR antagonist	5	Y	N	N	TST
(-)-MK-801	Non-competitive NMDAR antagonist	4	Y	N	N	TST
NENK	Non-competitive NMDAR antagonist	8	Y	N	N	Both
NVP-AAM077	Non-competitive NMDAR antagonist	1	N	N	N	FST
Arcaine	Competitive NMDAR antagonist	1	N	N	N	TST
CGP-37849	Competitive NMDAR antagonist	5	Y	N	N	FST
CPP	Competitive NMDAR antagonist	1	N	N	N	FST
BMS-986169	GluN2B antagonist	4	Y	N	N	FST
Eliprodil	GluN2B antagonist	8	Y	N	N	Both
Ifenprodil	GluN2B antagonist	5	Y	N	N	FST
Ro-25-6981	GluN2B antagonist	3	Y	N	N	Both
Traxoprodil	GluN2B antagonist	7	Y	N	N	FST
L-701,324	NMDA glycine receptor antagonist	6	Y	N	N	FST
Lanicemine	NMDA channel blocker	3	Y	N	N	TST
Acamprosate	NMDAR antagonist, GABAA upregulator	5	Y	N	N	TST
2-MeO-NEK	Novel NMDAR antagonist	8	Y	N	N	Both
4-MeO-NEK	Novel NMDAR antagonist	8	Y	N	N	Both
(2R,6R)-HNK	Ketamine metabolite	1	Y	N	N	FST
(2S,6S)-HNK	Ketamine metabolite	1	Y	N	N	FST
(R)-NK	Ketamine metabolite	1	Y	N	N	FST
(S)-NK	Ketamine metabolite	1	Y	N	N	FST
Dehydronorketamine	Ketamine metabolite	1	N	N	Y	FST
Norketamine	Ketamine metabolite	3	Y	N	N	FST

a The number of replicates included within the analysis with a replicate being defined as a single dose at a single time point from within a study (e.g. a four-dose acute study within a single paper would be classed as four replicates).

b The availability of data for each time point alongside which behavioural despair test data is available from.

### Data extraction/outcome measures

In addition to bibliographic information, the details of mouse strain, weight, age and housing were recorded from each study. Data about the pharmacological manipulation used were then collated and this included pre-treatment time, dosage, route of administration and compound used. Immobility time in the FST and/or TST was recorded in addition to noting the presence or absence of a pre-test session. Studies not measuring immobility time were excluded. If studies included data on locomotion or the NMDAR antagonist pharmacokinetics, this was also recorded. For locomotor data, only the method used and results found were recorded with the pre-treatment time not noted. Where possible, the mean value and standard deviation were extracted from within text or tables, but where this was not possible, data were reconstructed from graphs. If the extracted data for a study were not sufficient, we did not contact the original authors and instead excluded that data. For all measures, the number of animals per experiment was recorded; however, in circumstances where this was not clear, the lowest reported n number was used. In cases where studies did not report an n number, then the study was excluded (n = 2).

### Analysis

A meta-analysis was conducted using the Der Simonian and Laird random-effects model, utilising the Comprehensive Meta-Analysis (v3.0) software package (Biostat, USA). Data from both the FST and TST were analysed together (although test information was included in the forest plots) due to them having a similar model construct (Slattery and Cryan, 2014). Individual effects sizes were calculated for each study. To increase clarity, only the dose inducing the maximum effect size for any reported study is included in the main figures with the effect sizes for all doses and pre-treatment times included in Supplementary Material. Data are presented in separate forest plots based on pre-treatment times of ⩽60 min, 24 h and >24 h. Individual drugs, doses and pre-treatment times are indicated in the figures. Mean effect sizes were calculated and are included in the illustrations where data for four or more studies were available.

For each drug and pre-treatment time, heterogeneity was calculated using I^2^ with ranges of >50% considered as low, 50%–75% as moderate and >75% as high heterogeneity (Vesterinen et al., 2014). In order to assess the state of the field, all studies were also evaluated for risk of bias using the SYRCLE’s risk of bias tool for animal studies (Hooijmans et al., 2014). These metrics describe how well the methods were described (e.g. reporting of blinding and/or randomisation) as opposed to being able to judge the quality of the underlying study. Any sub-clinical doses of ketamine (⩽0.1 mg/kg) as well as anaesthetic ketamine doses (>30 mg/kg) were excluded from the meta-analysis.

Publication bias was assessed using funnel plots and Egger’s intercept was calculated (Egger et al., 1997), the more asymmetric funnel plots indicated a higher chance of publication bias. Exclusion of outliers only resulted in increased heterogeneity and had little to no impact on the effect sizes; therefore, outliers were not removed. Outlier detection was carried out using the interquartile range (IQR) method whereby the IQR, quartiles 1 (Q1) and 3 (Q3) were calculated and used such that data (x) not fitting within the range Q1 − (1.5 × IQR) ⩽ x ⩽ Q3 + (1.5 × IQR) was excluded. Due to high heterogeneity of data, no Duval and Tweedie trim and fill plots were used (Duval and Tweedie, 2000). Attempt to compensate for heterogeneity by performing meta-regression did not output any statistically significant results. Output graphics were constructed in GraphPad Prism 9 (GraphPad, USA), Tableau Software (Salesforce, USA) and Stata/MP 16 (StataCorp, USA).

## Results

### Reduced immobility time in mouse models of behavioural despair following acute (<60 min) pre-treatment with different NMDAR antagonists or the ketamine metabolite, HNK

**Figure 2.**
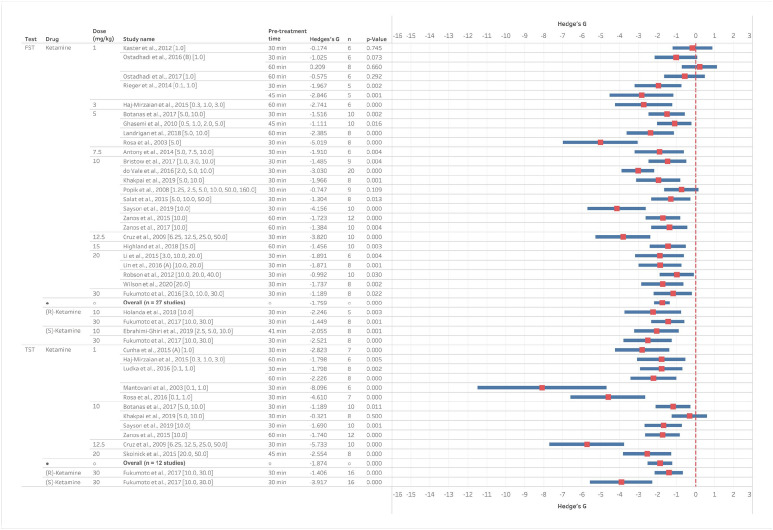
Ketamine reduces immobility time in the FST and TST in mice following acute administration and testing up 60-min post-treatment. Data are divided into studies using the FST or TST and Hedge’s G is plotted for the dose inducing the maximum effect size out of all the doses reported. The full-dose range available is indicated in the study name column and the dose illustrated in the graph is given in the second column. The n number for the study, pre-treatment time and statistical findings are also included. Negative Hedge’s G-value indicate increased immobility time following administration of drug compared to vehicle while positive values indicate decreased immobility time.

The acute effects of ketamine on behavioural despair in normal animals were reported in 28 studies using the FST and 12 studies using the TST. Similar reductions in immobility time were observed for both tasks and racemic ketamine with similar overall effects size of −1.76 (FST) and −1.87 (TST) (Figure 2). Results were consistent across studies for the different doses tested with most studies (3 of 4) finding 1 mg/kg did not significantly reduce immobility in the FST, but all studies for this dose found a significant reduction in the TST. Between doses of 3 and 30 mg/kg, all studies except one (24 of 25 studies) reported significant reduction in immobility for the FST and only one study did not report a reduction in immobility in the TST (7 of 8 studies reported reduced immobility). Studies testing the two stereoisomers of ketamine found similar reductions in immobility for R-ketamine and S-ketamine in both the FST and TST at 10 and 30 mg/kg.

**Figure 3.**
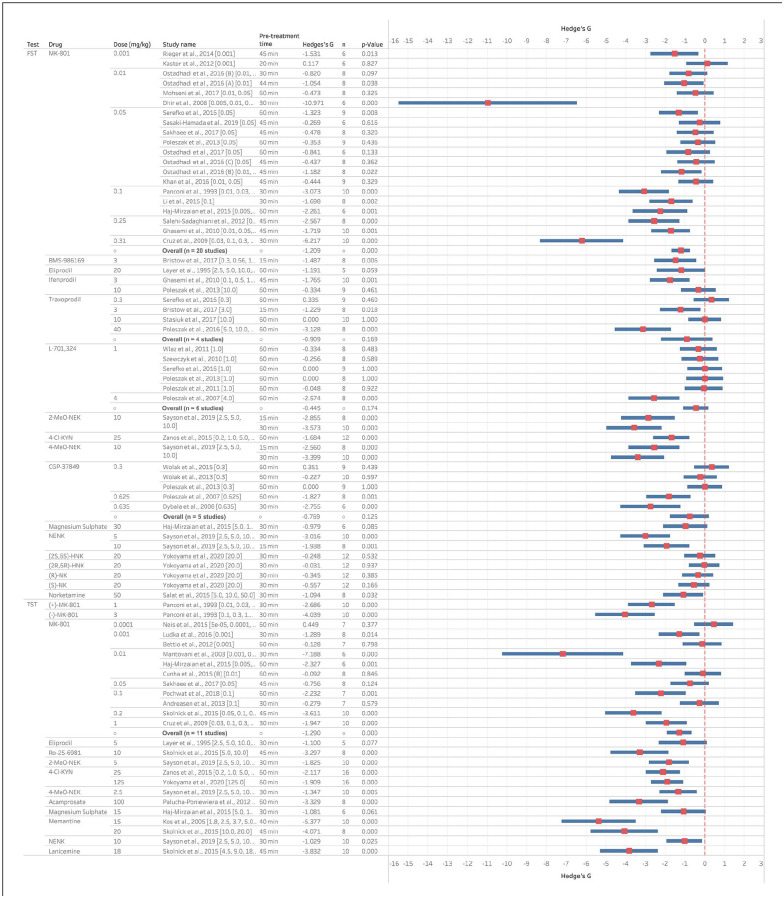
Non-ketamine NMDAR antagonists reduce immobility time in the FST and TST in mice following acute administration and testing up 60-min post-treatment. Data are divided into studies using the FST or TST and Hedge’s G is plotted for the dose inducing the maximum effect size out of all the doses tested and reported in this article. The full-dose range available is indicated in the study name column and the dose illustrated in the graph is given in the second column. The n number for the study, pre-treatment time and statistical findings are also included. Negative Hedge’s G value indicate increased immobility time following administration of drug compared to vehicle while positive values indicate decreased immobility time.

MK801 was the non-ketamine NMDAR antagonist most widely tested in pre-clinical models and results from both the FST and TST report similar reductions in immobility time following acute treatment (Figure 3). Between doses of 0.001 and 0.05 mg/kg, results were mixed with some finding a decrease but others not and this was consistent across both the FST and TST. Doses of 0.1 mg/kg or higher were more consistent with most studies (9 of 10) reporting reductions in immobility. Overall, effects sizes for MK801 in the FST were −1.21 and −1.29 for the TST. The results for the other NMDAR antagonists were more variable, but this may in part be due to the more limited numbers of studies which have been undertaken. There is tentative evidence that GluN2B antagonism can show clinical benefit (Preskorn et al., 2008), but results from the FST and TST following acute administration were mixed. There was only enough data to calculate an overall effect size for traxoprodil in the FST; however, the results were not significant (p = 0.17). There was also an impact of dose, with effects seen at higher doses (range = 0.3–40 mg/kg) which may not relate to specific engagement with the GluN2B containing NMDAR receptors (the effect concentration 50 has been reported to be 1 mg/kg in mice for prevention of NMDA-mediated CFos expression (Chenard et al., 1995, see Figure 3)). Two other NMDAR antagonists which have been tested clinically are lanicemine and memantine (Sanacora et al., 2017; Zarate et al., 2006). In the TST, both compounds reduced immobility time following acute administration with a large effect size (Figure 3). Studies testing competitive NMDAR antagonists (arcaine, CGP-37849 and CPP) or the NMDAR glycine site antagonist L-701,324 failed to detect an overall effect in either the FST or TST, but studies with other non-competitive NMDAR antagonists largely found reductions in immobility time (Figure 3).

The study selection criteria did not specifically aim to identify publications which had tested the ketamine metabolites however, following the publication by Zanos et al. (2016) several papers which had tested ketamine metabolites in either the FST or TST were identified. From those studies which did pass selection, only one study using norketamine reported a reduced immobility time (Sałat et al., 2015) whereas the other study testing either norketamine or HNK analogues did not show significant changes in immobility time compared to vehicle (Yokoyama et al., 2020).

### Ketamine and other NMDAR antagonists reduce immobility time in the FST and TST 24 h after administration

**Figure 4.**
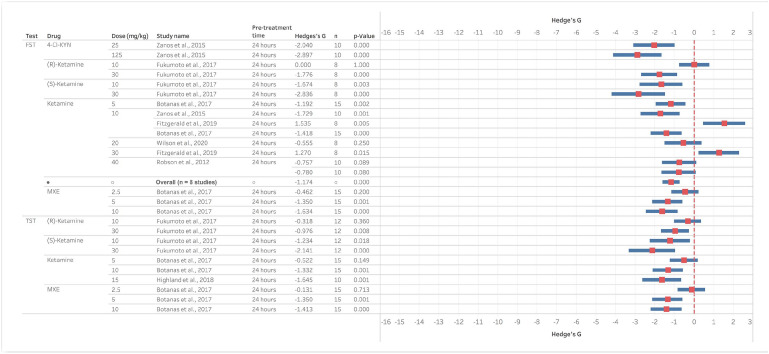
Ketamine and other NMDAR antagonists reduce immobility time in the FST and TST 24 h post-treatment. Data are divided into studies using the FST or TST and Hedge’s G is plotted for the dose inducing the maximum effect size out of all the doses tested and reported in this article. The full-dose range available is indicated in the study name column and the actual dose illustrated in the graph is given in the second column. The n number for the study, pre-treatment time and statistical findings are also included. Negative Hedge’s G value indicate increased immobility time following administration of drug compared to vehicle while positive values indicate decreased immobility time.

**Figure 5.**
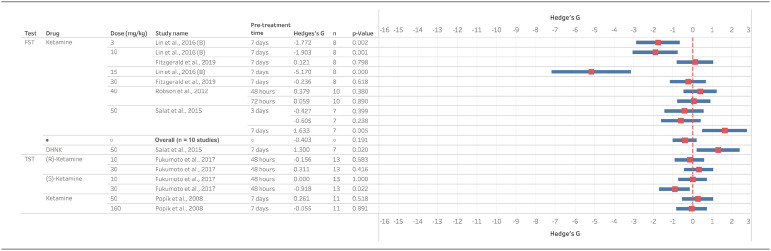
Ketamine and other NMDAR antagonists reduce immobility time in the FST and TST greater than 24-h post-treatment. Data are divided into studies using the FST or TST and Hedge’s G is plotted for the dose inducing the maximum effect size out of all the doses tested and reported in this article. The full-dose range available is indicated in the study name column and the actual dose illustrated in the graph is given in the second column. The n number for the study, pre-treatment time and statistical findings are also included. Negative Hedge’s G value indicate increased immobility time following administration of drug compared to vehicle while positive values indicate decreased immobility time.

Despite the antidepressant effects of ketamine having been linked to its ability to induce sustained changes in mood beyond the initial pharmacological effects of the drug, relatively a few studies have investigated the effects of different NMDAR antagonists at these longer time points. Only studies testing non-competitive antagonists were found for behavioural tests carried out 24 h after treatment (Figure 4) or 48 h – 7 days post-treatment (Figure 5). At 24-h post-ketamine, four of six studies reported a significant reduction in immobility time in the FST and the one study in the TST found significant effects at 10 but not 5 mg/kg. In the FST, S- but not R-ketamine reduced immobility time at 10 mg/kg but both had significant effects at 30 mg/kg with the same profile of efficacy seen in the TST. The non-competitive antagonists MXE and 4-Cl-KYN also reduced immobility time in both assays.

Only ketamine and the metabolite DHNK have been tested at time points longer than 24 h. The racemic mixture of ketamine has been reported to maintain efficacy at 7 days post-treatment in one study where 3, 10 and 15 mg/kg doses all significantly reduced immobility time in the FST (Lin et al., 2016b), but no effects were seen in a different study using 10 and 30 mg/kg (Fitzgerald et al., 2019). Higher doses have also been tested with one study finding effects at 7 but not 3 days post 50 mg/kg (Sałat et al., 2015), but a study testing 40 mg/kg found no effects 48 h or 7 days post-treatment (Robson et al., 2012). Comparing the S- versus R-isoforms, S-ketamine at 30 but not 10 mg/kg reduced immobility in the TST and no effects were found for the R-isomer when measured 24 h after administration. The one study testing the metabolite DHNK found significant effects on immobility time in the FST at 50 mg/kg 7 days post-treatment (Sałat et al., 2015).

### Effects of NMDAR antagonists upon locomotor activity

**Figure 6.**
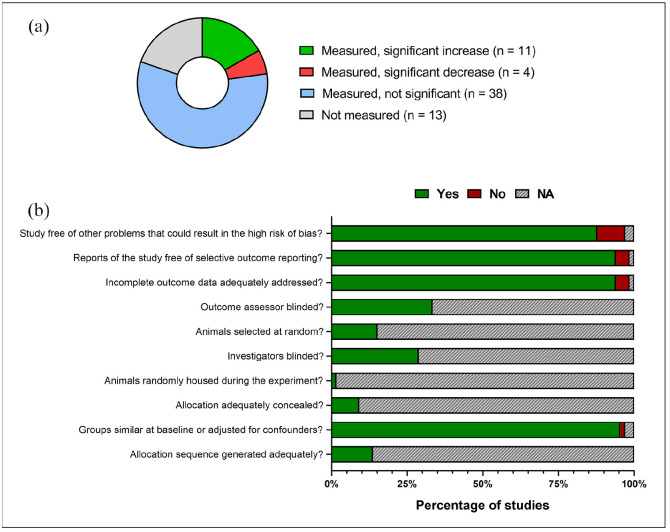
Locomotor effects of treatment in the FST/TST and measures of study quality. (a) Pie chart showing the number of studies reporting locomotor outcomes following treatment with NMDAR antagonists in the FST or TST. (b) Summary of study quality according to the SYRCLE reporting guidelines (Hooijmans et al., 2014) showing the percentage of included studies that either complied with each item or did not report.

80.3% of studies reported the effects of an NMDAR antagonist on locomotor activity (Figure 6(a)). Thirteen studies out of a total of 66 either did not measure changes in locomotion or did not provide data for the groups of interest. Out of the studies that did measure locomotor activity, 38 reported no significant change, 11 reported a significant increase and 4 studies reported a significant decrease in locomotion. The most frequently tested doses for ketamine were 1 and 10 mg/kg which occurred in eight studies and 0.05 mg/kg MK-801 was examined in 11 studies.

### Pharmacokinetic data

Only one study investigated the underlying pharmacodynamics and two studies have measured drug levels in plasma, for results of these findings please refer to Table 2.

**Table 2.** Summary of papers reporting pharmacokinetic data for BMS-986169, (R)-ketamine and (S)-ketamine.

Drug	Study	Dose (mg/kg)	Time after dosing	Administration route	Concentration (µM)^a^			% GluN2B occupancy
					Plasma	Brain tissue	CSF	
(R)-Ketamine	Fukumoto et al. (2017)	10	30 min	i.p.	0.88 ± 0.35	2.15 ± 0.76	0.24	–
(R)-Ketamine	Fukumoto et al. (2017)	10	24 h	i.p.	<LLOQ	<LLOQ	<LLOQ	–
(R)-Ketamine	Fukumoto et al. (2017)	30	30 min	i.p.	4.03 ± 0.65	10.34 ± 1.62	1.90	–
(R)-Ketamine	Fukumoto et al. (2017)	30	24 h	i.p.	<LLOQ	<LLOQ	<LLOQ	–
(S)-Ketamine	Fukumoto et al. (2017)	10	30 min	i.p.	1.58 ± 0.14	2.22 ± 0.35	0.53	–
(S)-Ketamine	Fukumoto et al. (2017)	10	24 h	i.p.	<LLOQ	<LLOQ	<LLOQ	–
(S)-Ketamine	Fukumoto et al. (2017)	30	30 min	i.p.	7.45 ± 0.85	9.71 ± 1.70	1.39	–
(S)-Ketamine	Fukumoto et al. (2017)	30	24 h	i.p.	<LLOQ	0.82 ± 0.792	<LLOQ	–
BMS-986169	Bristow et al. (2017)	0.3	25 min	i.v.	0.091 ± 0.037	0.29 ± 0.115	–	41.9 ± 3.4^b^
BMS-986170	Bristow et al. (2017)	0.56	25 min	i.v.	0.094 ± 0.052	0.32 ± 0.183	–	65.5 ± 7.2^b^
BMS-986171	Bristow et al. (2017)	1	25 min	i.v.	0.27 ± 0.128	0.75 ± 0.215	–	73.3 ± 2.3^b^
BMS-986172	Bristow et al. (2017)	3	25 min	i.v.	0.76 ± 0.332	1.64 ± 0.586	–	92.3 ± 3.7^b^

LLOQ: lower limit of quantification (between 0.4 and 8.4 nM depending upon multiple factors); CSF: cerebral spinal fluid; i.p.: intraperitoneal; i.v.: intravenous.

a Data are shown as mean value ± SD.

b Tested after 15 min.

### Heterogeneity

The I^2^ test indicated moderate to high levels of heterogeneity (see Supplementary Figure 2.3): moderate for acute ketamine (I^2^ = 71.88%) and acute MK-801 (I^2^ = 73.93%), high for acute other NMDAR antagonists (I^2^ = 80.57%); 24-h data for all NMDAR antagonists were moderately heterogeneous (I^2^ = 73.47%) and highly heterogeneous after 24 h (I^2^ = 75.99%).

### Study quality and publication bias

Only 33.3% of studies explicitly stated that the outcome assessor was blinded and less than 15.2% confirmed that animals were selected at random; 1.5% of the studies housed animals randomly (see Supplementary Table 3.1). Most studies included details of methods ensuring homogeneity of sample groups as well as addressing incomplete data without resorting to selective outcome reporting (see Figure 6(b) for a summary). There was evidence for publication bias of data from all drugs at all pre-treatment times (see Supplementary Figures 2.1 and 2.2 alongside Supplementary Table 2.3).

## Discussion

The RAADs are distinct from conventional antidepressants in their ability to induce both immediate antidepressant effects and efficacy which extends beyond their initial pharmacological effects. Rodent models of behavioural despair are the most commonly used behavioural readout of these antidepressant effects, but these tests have been criticised as being limited to predictive validity for drugs from certain pharmacological classes such as monoaminergic re-uptake or enzyme inhibitors (Berton et al., 2012). Despite the fact studies reporting efficacy of NMDAR antagonists in rodent models of behavioural despair were reported in 1992, it was not until 2000 that ketamine was tested and found to be effective in patients (Maj et al., 1992) possibly reflecting this lack of confidence in interpreting rodent data from these tests. In this review of publications reporting the effects of NMDAR antagonists and related compounds in mouse models of behavioural despair, we find that most studies report similar positive effects following acute administration (behavioural testing <60 min post-treatment). Acute effects of ketamine were not seen at higher doses, potentially due to ketamine’s sedative properties causing increased immobility. Evidence that the FST and/or TST can predict the sustained effects of RAADs from this class was less robust with both a mixture of positive and negative findings being observed in the literature. There was also little consistency in the effects of the same dose when reported in different studies.

Although ketamine is a non-competitive NMDAR antagonist, it also interacts with other receptors at clinical doses and it has been hypothesised that its effects are mediated by its metabolite, HNK acting directly on AMPA receptors (Aleksandrova et al., 2017). One reason why studies have sought to investigate alternative targets from the NMDAR in mediating ketamine’s RAAD effects has been the lack of efficacy of other NMDAR antagonists tested in clinical trials. Despite both lanicemine and memantine acting as antagonists at the NMDAR, clinical trials have failed to find evidence of any RAAD effects (Sanacora et al., 2017; Zarate et al., 2006). It should be noted, however, that early stage clinical trials can also have many of the same limitations seen in pre-clinical studies (e.g. small samples sizes) meaning that RAAD efficacy may be overstated or not found. Interestingly this contrasts with the effects found in these rodent models where most studies found significantly reduced immobility time predictive of an antidepressant effect (Figure 3). The opposite outcomes, however, are seen when comparing the effects of the GluN2B-specific NMDAR antagonists of which traxoprodil has shown promising early clinical results (Preskorn et al., 2008). However, in the FST and TST, most studies failed to find effects on immobility time, although it should be noted that a few studies have investigated the effects at the longer time points post-treatment. These results contrast to some alternative methods such as the judgement bias test which found no effect of lanicemine or memantine upon animals’ interpretation of an ambiguous cue whereas ketamine and the NR2B selective NMDAR antagonist, traxoprodil caused a positive bias towards this ambiguous cue (Hales et al., 2020). Data for the judgement bias task (JBT) are also more consistent with clinical efficacy when compared to the sucrose preference test (SPT), where although lanicemine shows no efficacy following chronic social defeat stress (Qu et al., 2017), mematine was found to reverse decreased sucrose preference following chronic corticosterone administration (Li et al., 2020). Traxoprodil has not yet been assessed in the SPT.

Studies investigating effects of (S)-ketamine and (R)-ketamine enantiomers have found (S)-ketamine to be four times more potent at the NMDAR than (R)-ketamine and so would be expected to display much stronger antidepressant effects. However, the results of three rodent depression tests, including the FST, indicated that (R)-ketamine exhibits greater antidepressant potency (Zanos et al., 2016). Based on the data reviewed here, there was no obvious relationship between the relative potency of the different enantiomers in the behavioural despair models and their NMDAR affinity. Several studies identified in the search had looked at the effects of the ketamine metabolites. These compounds are thought to exert their effects through a non-NMDAR mechanism and the papers we reviewed found no evidence of efficacy in the behavioural despair models. Our search was not designed to identify papers specifically testing the ketamine metabolites and so provides an incomplete record of this literature and interpretation is therefore limited.

Based on the studies reviewed in this analysis, the FST and TST do not reliably predict the clinical efficacy of RAADs. It should, however, be noted that these findings may not generalise to studies using depression disease models or those using rats. Although not included in this analysis, RAAD effects have also been found clinically with the psychedelic psilocybin. Data from both wild-type mice (Mahmoudi et al., 2018) and selectively bred rat lines (Jefsen et al., 2019) do not mirror clinical effects. It is worth, however, noting findings from Hibicke et al. (2020) who reported a decreased FST immobility time in the Wistar–Kyoto rats following a 35-day pre-treatment period. One of the potential confounds with the FST and TST is their sensitivity to locomotor effects (Porsolt et al., 1977; Slattery and Cryan, 2014). Only 25% of studies included a test of effects on locomotion but those which did found a mixture of results suggesting there may be locomotor effects at doses which are also antidepressant. Changes in locomotor activity have previously been reported for NMDAR antagonists (Irifune et al., 1991; Liljequist et al., 1991), including at doses commonly tested in relation to predicting antidepressant effects but only 75% of studies had included a measure of locomotor activity (Danysz et al., 1994). Another consideration is the extent to which a normal mouse provides a relevant model for testing antidepressant effects. We limited our review to studies in non-stressed mice; however, a previous review of this literature suggested that efficacy was more variable in non-stressed animals (Polis et al., 2019) and, based on their review of the literature and their own studies in stressed versus non-stressed mice, these authors propose that ketamine does not have robust antidepressant-like properties in unstressed animals. Finally, the dose of drugs used is relatively high in rodents relative to humans and a few studies (n = 2) actually measured the plasma concentration following administration. This means that a further limitation of these studies in terms of predicting efficacy is whether the dose being tested is within a clinically relevant plasma concentration therefore having similar levels of receptor occupancy. For example, it has been reported that the maximal plasma concentration following a typical 40-min infusion in humans is 0.78 µM, lower than the plasma concentrations (0.9–1.58 µM) reported in mice by Fukumoto et al. (2017) (see Table 2) at 10 mg/kg i.p. Many studies in mice use higher doses still with plasma concentrations at 30 mg/kg i.p. being as high as 7.45 µM (Fukumoto et al., 2017). Future studies would benefit from careful consideration of the animal depression model used alongside choosing doses of antidepressant compound that mirror as closely as possible the known pharmacokinetics in humans.

It should also be noted that the results were limited by the data availability and high heterogeneity. For example, MK-801 data 24-h post-treatment would have been useful since FST and TST results of other NMDAR antagonists suggested antidepressant effects and another study suggested that ketamine shows more antidepressant efficacy compared to other NMDAR antagonists after 24 h, while MK-801 has been found to produce no significant effect in FST 24-h post-treatment (Zanos et al., 2016; Zanos and Gould, 2018). Unfortunately, not enough data were available to compare effects of ketamine and other NMDAR antagonists at 7 days pre-treatment, these data could be a useful contributor to better understanding the role of NMDAR antagonism in ketamine’s long-term antidepressant effects and translational validity. One of the other common problems found across the studies in this review was the lack of details relating to the use of methodology to reduce bias, such as randomisation and blinding although most studies did not report this and so it is not known if these experiment design approaches were included or not. Many details, often overlooked, can have a significant impact upon psychopharmacology studies which were captured in the risk of bias assessment. Factors such as group versus single housing (Karolewicz and Paul, 2001), different baseline characteristics such as strain, sex and age (Bogdanova et al., 2013) alongside inconsistencies in scoring methodologies (Borsini and Meli, 1988) and a lack of power calculations leading to underpowered studies (Smallheiser et al., 2021) have all been found to impact results in the FST and TST. Finally, other issues such as not being explicit with inclusion/exclusion criteria and unknown group allocation methodologies all detract from the ability to interpret the data. Due to this unavailability of data, it is not possible to be certain that if all studies contained in this review were performed to an appropriate standard that the conclusions of our analysis would be the same as presently reported. Analysis of publication bias suggested a high probability that studies which investigated NMDAR antagonism in FST and TST and produced negative data were considered less relevant and were not published. This could mean that the effects of some NMDAR antagonists are over-represented and therefore so is the significance of their effect. This is, however, in no way unique to studies involving NMDARs in models of behavioural despair but is a pervasive problem across all science (Fanelli, 2012).

Concerns relating to the poor predictive validity, weak translational relevance alongside ethical concerns associated with use of the FST and TST have long been recognised within the field of depression research (Molendijk and de Kloet, 2015; Reardon, 2019; Sewel et al., 2021; Slaney et al., 2018; Slattery and Cryan, 2012). Efforts have been made to translate the tasks used to assess depression symptomology in humans into rodents to provide tasks for use in animal research that show greater face and construct validity than the FST/TST. One such example is the affective bias task (ABT) where animals bias learning due to affective state changes caused by either pharmacological or psychosocial manipulations (Stuart et al., 2013). This task shows strong predictive validity for a range of delayed-onset and rapid-acting antidepressants alongside pro-depressant manipulations (Hinchcliffe et al., 2017; Stuart et al., 2013, 2015, 2019, 2017). As previously mentioned, the JBT (Neville et al., 2020) has similarly showed predictive validity when tested with a range of pro-depressant manipulations alongside antidepressants compounds where crucially the therapeutic lag seen in the clinic is also apparent in animals tested in the JBT after being given either delayed-onset or rapid-acting antidepressants (Hales et al., 2016, 2017, 2020, 2021). The probabilistic reward task (PRT) is another widely used task in man (Pizzagalli 2014) that has been successfully translated into both rodents (Kangas et al., 2020) and marmosets (Wooldridge et al., 2021) where the RAADs scopolamine and ketamine increase the bias that reward has upon interpretation of an ambiguous cue. These tasks show huge improvements over the FST/TST in terms of predictive, construct and face validity while also being much lower stress for animals. They do suffer from the drawback of taking significantly longer to both train animals in and assess putative antidepressant compounds in. One potential solution to this is from the suggestion that translational biomarkers such as Brain derived neurotrophic factor (BDNF) should be used for initial pre-clinical screening (Sewel et al., 2021). Measurement of biomarkers such as increased BDNF from either blood (Brunoni et al., 2008; Duman and Aghajanian, 2014) or release from cultured neurones (Lepack et al., 2016) could be a vital first screening step for any putative antidepressant compound before investment is made in testing in a translational task such as those previously described.

In summary, this analysis suggests that rodent models of behavioural despair are not predictive of clinical efficacy of drugs acting via NMDARs and their use for studies into the mechanisms underlying ketamine’s clinical effects in treatment-resistant depression may be limited when using normal mice. There is a lack of consistency in findings with most drugs except ketamine. NR2B selective antagonists which have shown clinical promise did not induce reliable changes in immobility time in mice and the NMDAR antagonists memantine and lanicemine did reduce immobility time in these models despite their lack of clinical efficacy (Sanacora et al., 2017; Zarate et al., 2006). We, therefore, suggest that the FST and TST are not suitable assays for the assessment of rapid-onset antidepressant efficacy in rodents. Alternative methods which utilise both biomarkers and more translational paradigms may provide more reliable pre-clinical model, although these lack the simplicity of the FST and TST (Hales et al., 2017; Robinson, 2018; Sewel et al., 2021; Stuart et al., 2013, 2015).

## Supplemental Material

sj-pdf-1-bna-10.1177_23982128221081645 – Supplemental material for A systematic review of studies investigating the acute effects of N-methyl-D-aspartate receptor antagonists on behavioural despair in normal animals suggests poor predictive validity Click here for additional data file. Supplemental material, sj-pdf-1-bna-10.1177_23982128221081645 for A systematic review of studies investigating the acute effects of N-methyl-D-aspartate receptor antagonists on behavioural despair in normal animals suggests poor predictive validity by Martin Viktorov, Matthew P. Wilkinson, Victoria C. E. Elston, Medi Stone and Emma S. J. Robinson in Brain and Neuroscience Advances
