# Supplementary material for: A systematic review of studies investigating the acute effects of N-methyl- D -aspartate receptor antagonists on behavioural despair in normal animals suggests poor predictive validity
Source: Brain Neurosci Adv. 2022 Mar 12;6:23982128221081645. doi: 10.1177/23982128221081645 (PMC8922211; doi:10.1177/23982128221081645)
Supplement: sj-pdf-1-bna-10.1177_23982128221081645 – Supplemental material for A systematic review of studies investigating the acute effects of N-methyl-D-aspartate receptor antagonists on behavioural despair in normal animals suggests poor predictive validity [file sj-pdf-1-bna-10.1177_23982128221081645.pdf]

Supplementary Data

Forest plots

SF1.1 Ketamine forest plot – all doses 2
SF1.2 MK-801 forest plot – all doses 3
SF1.3 Other NMDAR antagonists – all doses 4

Publication bias and heterogeneity

SF2.1 Acute, 24h, 24h+ funnel plot 5
SF2.2 Summary funnel plots 6
ST2.3 Heterogeneity and publication bias estimates for each drug at different pre-treatment times 7

List of studies

ST3.1 Included studies with measures of bias 8

Test	Drug	Dose (mg/kg)	Study name	Pre-treatment time	Hedges's G	n	p-Value
FST	(R)-Ketamine	10	Holanda et al., 2018	30 min	-1.517	5	0.022
					-2.246	5	0.003
			Fukumoto et al., 2017	24 hours	0.000	8	1.000
				30 min	-0.913	8	0.028
		30	Fukumoto et al., 2017	24 hours	-1.776	8	0.000
				30 min	-1.449	8	0.001
	(S)-Ketamine	2.5	Ebrahimi-Ghiri et al., 2019	41 min	-0.864	8	0.082
		5	Ebrahimi-Ghiri et al., 2019	41 min	-1.413	8	0.008
		10	Fukumoto et al., 2017	24 hours	-1.674	8	0.003
				30 min	-1.720	8	0.002
			Ebrahimi-Ghiri et al., 2019	41 min	-2.055	8	0.001
		30	Fukumoto et al., 2017	24 hours	-2.836	8	0.000
				30 min	-2.521	8	0.000
	Ketamine	0.1	Rieger et al., 2014	45 min	-1.549	6	0.012
		1	Rieger et al., 2014	30 min	-1.967	5	0.002
				45 min	-2.846	5	0.001
			Ostadhadi et al., 2017	60 min	-0.575	6	0.292
			Ostadhadi et al., 2016 (B)	30 min	-1.025	6	0.073
				60 min	0.209	8	0.660
			Kaster et al., 2012	30 min	-0.174	6	0.745
			Haj-Mirzaian et al., 2015	60 min	-0.989	6	0.083
			Ghasemi et al., 2010	45 min	-0.452	10	0.298
			Bristow et al., 2017	30 min	-0.393	9	0.386
		2.5	Popik et al., 2008	30 min	0.335	9	0.460
		3	Lin et al., 2016 (B)	7 days	-1.772	8	0.002
			Li et al., 2015	30 min	-0.477	6	0.379
			Haj-Mirzaian et al., 2015	60 min	-2.741	6	0.000
			Fukumoto et al., 2016	30 min	0.106	8	0.823
			Bristow et al., 2017	30 min	-1.303	9	0.009
		5	Salat et al., 2015	30 min	-0.116	8	0.806
			Rosa et al., 2003	30 min	-5.019	8	0.000
			Popik et al., 2008	30 min	-0.718	9	0.122
			Landrigan et al., 2018	60 min	-2.385	8	0.000
			Khakpai et al., 2019	30 min	-0.074	8	0.875
			Ghasemi et al., 2010	45 min	-1.111	10	0.016
			do Vale et al., 2016	30 min	-1.628	17	0.000
			Botanas et al., 2017	24 hours	-1.192	15	0.002
				30 min	-1.516	10	0.002
			Antony et al., 2014	30 min	-0.864	6	0.124
		10	Zanos et al., 2017	60 min	-1.384	10	0.004
			Zanos et al., 2015	24 hours	-1.729	10	0.001
				60 min	-1.723	12	0.000
			Sayson et al., 2019	30 min	-1.067	8	0.036
					-1.846	8	0.001
					-4.156	10	0.000
			Salat et al., 2015	30 min	-1.304	8	0.013
			Robson et al., 2012	30 min	-0.813	10	0.069
			Popik et al., 2008	30 min	-0.747	9	0.109
			Lin et al., 2016 (B)	7 days	-1.903	8	0.001
			Lin et al., 2016 (A)	30 min	-0.732	6	0.186
					-1.643	8	0.003
			Li et al., 2015	30 min	-0.889	6	0.114
			Landrigan et al., 2018	60 min	-1.324	8	0.012
			Khakpai et al., 2019	30 min	-1.966	8	0.001
			Fukumoto et al., 2016	30 min	-0.463	8	0.334
			Fitzgerald et al., 2019	7 days	0.121	8	0.798
				24 hours	1.535	8	0.005
			do Vale et al., 2016	30 min	-3.030	20	0.000
			Bristow et al., 2017	30 min	-1.485	9	0.004
			Botanas et al., 2017	24 hours	-1.418	15	0.000
				30 min	-1.389	10	0.004
			Antony et al., 2014	30 min	-1.729	6	0.007
		15	Lin et al., 2016 (B)	7 days	-5.170	8	0.000
			Highland et al., 2018	60 min	-1.456	10	0.003
		20	Wilson et al., 2020	24 hours	-0.555	8	0.250
				30 min	-1.737	8	0.002
			Robson et al., 2012	30 min	-0.992	10	0.030
			Lin et al., 2016 (A)	30 min	-1.576	8	0.004
					-1.739	8	0.002
					-1.871	8	0.001
			Li et al., 2015	30 min	-1.891	6	0.004
		25	Cruz et al., 2009	30 min	0.356	10	0.410
					-1.292	10	0.006
		30	Fukumoto et al., 2016	30 min	-1.189	8	0.022
			Fitzgerald et al., 2019	7 days	-0.236	8	0.618
				24 hours	1.270	8	0.015
		40	Robson et al., 2012	24 hours	-0.757	10	0.089
					-0.780	10	0.080
				30 min	-1.459	10	0.003
					-1.467	10	0.003
				48 hours	0.379	10	0.380
				72 hours	0.059	10	0.890
		50	Salat et al., 2015	3 days	-0.427	7	0.399
					-0.605	7	0.238
				7 days	1.633	7	0.005
				30 min	-2.358	8	0.000
			Popik et al., 2008	14 days	-1.193	9	0.015
				30 min	-3.413	9	0.000
			Cruz et al., 2009	30 min	-0.421	10	0.331
		0.5	Ghasemi et al., 2010	45 min	-0.064	10	0.882
		1.25	Popik et al., 2008	30 min	-0.542	9	0.237
		2	Ghasemi et al., 2010	45 min	-1.089	10	0.018
			do Vale et al., 2016	30 min	-1.175	14	0.001
		6.25	Cruz et al., 2009	30 min	-0.532	10	0.223
		7.5	Antony et al., 2014	30 min	-1.910	6	0.004
		12.5	Cruz et al., 2009	30 min	-3.820	10	0.000
TST	(R)-Ketamine	10	Fukumoto et al., 2017	24 hours	-0.318	12	0.360
				30 min	-0.731	16	0.040
				48 hours	-0.156	13	0.683
		30	Fukumoto et al., 2017	24 hours	-0.976	12	0.008
				30 min	-1.406	16	0.000
				48 hours	0.311	13	0.416
	(S)-Ketamine	10	Fukumoto et al., 2017	24 hours	-1.234	12	0.018
				30 min	-2.134	16	0.000
				48 hours	0.000	13	1.000
		30	Fukumoto et al., 2017	24 hours	-2.141	12	0.000
				30 min	-3.917	16	0.000
				48 hours	-0.918	13	0.022
	Ketamine	0.1	Rosa et al., 2016	30 min	0.354	7	0.483
			Mantovani et al., 2003	30 min	0.492	6	0.364
			Ludka et al., 2016	30 min	-0.335	8	0.482
			Cunha et al., 2015 (B)	30 min	0.334	8	0.483
				60 min	1.705	8	0.002
			Bettio et al., 2012	30 min	0.000	7	1.000
		0.3	Haj-Mirzaian et al., 2015	60 min	-0.959	6	0.091
		1	Rosa et al., 2016	30 min	-4.610	7	0.000
			Mantovani et al., 2003	30 min	-8.096	6	0.000
			Ludka et al., 2016	30 min	-1.798	8	0.002
				60 min	-2.226	8	0.000
			Haj-Mirzaian et al., 2015	60 min	-1.798	6	0.005
			Cunha et al., 2015 (A)	30 min	-0.985	7	0.065
					-2.823	7	0.000
		5	Khakpai et al., 2019	30 min	0.000	8	1.000
			Botanas et al., 2017	24 hours	-0.522	15	0.149
				30 min	-1.004	10	0.028
		10	Zanos et al., 2015	60 min	-1.740	12	0.000
			Sayson et al., 2019	30 min	-1.690	10	0.001
			Li et al., 2019	2 hours	0.141	8	0.766
			Khakpai et al., 2019	30 min	-0.321	8	0.500
			Cunha et al., 2015 (B)	75 min	0.837	8	0.091
			Botanas et al., 2017	24 hours	-1.332	15	0.001
				30 min	-1.189	10	0.011
		15	Highland et al., 2018	24 hours	-1.645	10	0.001
		20	Skolnick et al., 2015	45 min	-2.554	8	0.000
		50	Skolnick et al., 2015	45 min	-1.652	8	0.003
			Popik et al., 2008	7 days	0.261	11	0.518
				40 min	-2.057	11	0.000
			Cruz et al., 2009	30 min	0.293	10	0.497
		12.5	Cruz et al., 2009	30 min	-4.504	10	0.000
					-5.733	10	0.000
		160	Popik et al., 2008	7 days	-0.055	11	0.891

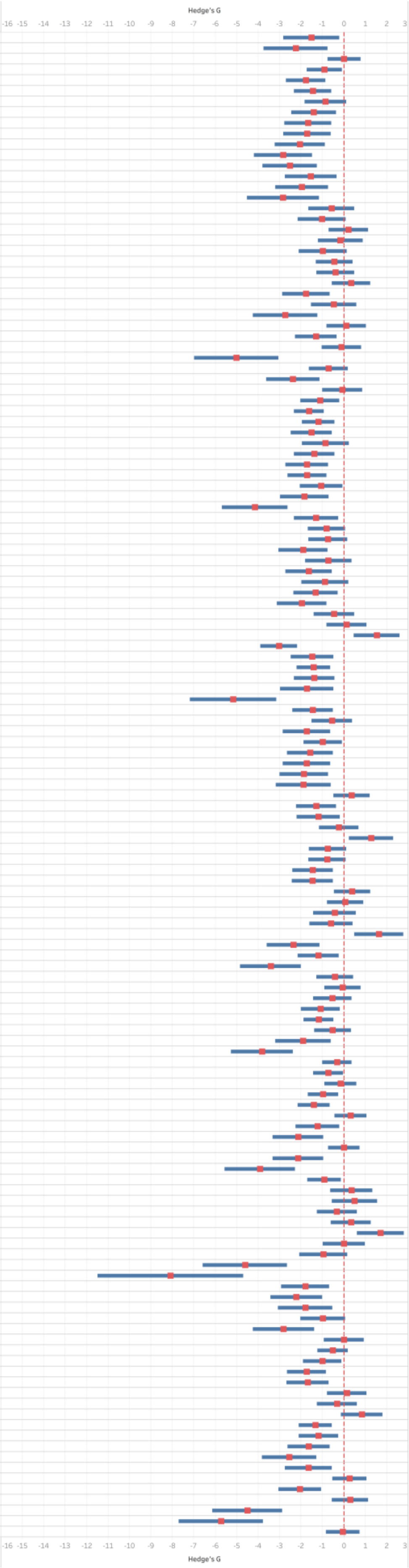

Supplementary figure 1.1 Forest plot showing effects of ketamine treatment in the FST and TST subdivided by dose and pre-treatment time

Test	Drug	Dose (mg/kg)	Study name	Pre-treatment time	Hedges's G	n	p-Value
FST	MK-801	0.001	Kaster et al., 2012	20 min	0.117	6	0.827
			Rieger et al., 2014	45 min	-1.531	6	0.013
		0.01	Dhir et al., 2008	30 min	-4.779	6	0.000
					-6.221	6	0.000
					-10.971	6	0.000
			Ghasemi et al., 2010	45 min	-0.465	10	0.285
			Khan et al., 2016	45 min	-0.302	9	0.504
			Mohseni et al., 2017	60 min	-0.473	8	0.325
			Ostadhadi et al., 2016 (A)	44 min	-1.054	8	0.038
			Ostadhadi et al., 2016 (B)	30 min	-0.820	8	0.097
			Panconi et al., 1993	30 min	-2.045	10	0.000
			Salehi-Sadaghiani et al., 2012	45 min	0.176	8	0.710
		0.05	Ghasemi et al., 2010	45 min	-0.214	10	0.618
			Haj-Mirzaian et al., 2015	60 min	-0.989	6	0.083
			Khan et al., 2016	45 min	-0.444	9	0.329
			Mohseni et al., 2017	60 min	-0.209	8	0.660
			Ostadhadi et al., 2016 (B)	45 min	-1.182	8	0.022
			Ostadhadi et al., 2016 (C)	45 min	0.000	8	1.000
					-0.437	8	0.362
			Ostadhadi et al., 2017	60 min	-0.841	6	0.133
			Poleszak et al., 2013	60 min	-0.353	9	0.436
			Sakhaee et al., 2017	45 min	-0.478	8	0.320
			Salehi-Sadaghiani et al., 2012	45 min	-0.334	8	0.484
			Sasaki-Hamada et al., 2019	45 min	-0.269	6	0.616
			Serefko et al., 2016	60 min	-1.323	9	0.008
		0.1	Cruz et al., 2009	30 min	-3.516	10	0.000
			Ghasemi et al., 2010	45 min	-1.199	10	0.010
			Haj-Mirzaian et al., 2015	60 min	-2.261	6	0.001
			Li et al., 2015	30 min	-1.437	8	0.007
					-1.570	8	0.004
					-1.698	8	0.002
			Panconi et al., 1993	30 min	-3.073	10	0.000
			Salehi-Sadaghiani et al., 2012	45 min	-1.728	8	0.002
		0.25	Ghasemi et al., 2010	45 min	-1.719	10	0.001
			Salehi-Sadaghiani et al., 2012	45 min	-2.567	8	0.000
		0.31	Cruz et al., 2009	30 min	-6.217	10	0.000
		0.005	Dhir et al., 2008	30 min	0.517	6	0.341
					0.471	6	0.385
					-0.755	6	0.174
		0.025	Dhir et al., 2008	30 min	-3.207	6	0.000
		0.03	Cruz et al., 2009	30 min	-0.843	10	0.060
					-1.845	10	0.000
			Panconi et al., 1993	30 min	-2.233	10	0.000
		○	**Overall (n = 43 studies)**	○	-1.263	○	0.000
TST	(+)-MK-801	0.01	Panconi et al., 1993	30 min	-0.037	10	0.931
		0.1	Panconi et al., 1993	30 min	-1.179	10	0.012
		0.3	Panconi et al., 1993	30 min	-1.801	10	0.000
		1	Panconi et al., 1993	30 min	-2.686	10	0.000
		0.03	Panconi et al., 1993	30 min	-0.530	10	0.225
		○	**Overall (n = 5 studies)**	○	-1.188	○	0.007
	(-)-MK-801	0.1	Panconi et al., 1993	30 min	-0.125	10	0.770
		0.3	Panconi et al., 1993	30 min	-1.033	10	0.024
		1	Panconi et al., 1993	30 min	-2.543	10	0.000
		3	Panconi et al., 1993	30 min	-4.039	10	0.000
		○	**Overall (n = 4 studies)**	○	-1.843	○	0.018
	MK-801	0.0001	Neis et al., 2015	60 min	0.449	7	0.377
		0.001	Bettio et al., 2012	60 min	-0.128	7	0.798
			Ludka et al., 2016	30 min	-1.289	8	0.014
			Mantovani et al., 2003	30 min	0.066	6	0.902
			Neis et al., 2015	60 min	0.175	7	0.727
		0.01	Cunha et al., 2015 (B)	60 min	-0.092	8	0.846
			Haj-Mirzaian et al., 2015	60 min	-2.327	6	0.001
			Mantovani et al., 2003	30 min	-7.188	6	0.000
		0.05	Sakhaee et al., 2017	45 min	-0.756	8	0.124
			Skolnick et al., 2015	45 min	1.011	10	0.031
		0.1	Andreasen et al., 2013	30 min	-0.279	7	0.579
			Cruz et al., 2009	30 min	-0.440	10	0.310
			Pochwat et al., 2018	60 min	-2.232	7	0.001
			Skolnick et al., 2015	45 min	-1.857	10	0.000
		0.2	Skolnick et al., 2015	45 min	-3.611	10	0.000
		0.3	Cruz et al., 2009	30 min	-0.137	10	0.749
		0.31	Cruz et al., 2009	30 min	-0.252	10	0.559
		1	Cruz et al., 2009	30 min	-1.947	10	0.000
		0.56	Cruz et al., 2009	30 min	-1.683	10	0.001
		5e-05	Neis et al., 2015	60 min	0.266	7	0.597
		0.0005	Neis et al., 2015	60 min	0.291	7	0.563
		0.005	Haj-Mirzaian et al., 2015	60 min	-0.699	6	0.205
		○	**Overall (n = 22 studies)**	○	-0.790	○	0.001

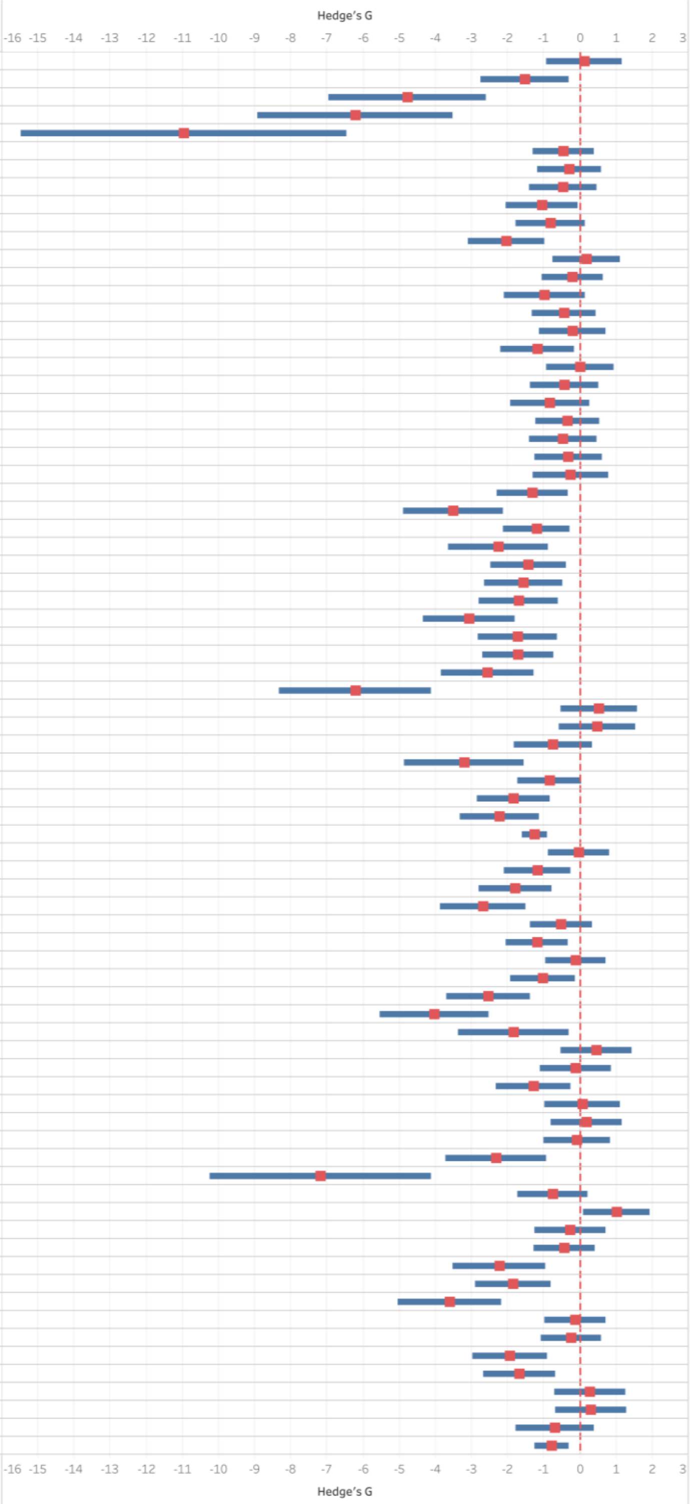

Supplementary figure 1.2 Forest plot showing effects of MK-801 treatment in the FST and TST subdivided by dose and pre-treatment time.

Test	Drug	Dose (mg/kg)	Study name	Pre-treatment time	Hedges's G	n	p-Value
FST	BMS-986169	0.3	Bristow et al., 2017	15 min	-0.780	8	0.113
		1	Bristow et al., 2017	15 min	-1.221	8	0.019
		3	Bristow et al., 2017	15 min	-1.487	8	0.006
		0.56	Bristow et al., 2017	15 min	-1.394	8	0.009
		○	**Overall (4 studies)**	○	-1.212	○	0.003
	Eliprodil	5	Layer et al., 1995	60 min	-0.626	5	0.287
		10	Layer et al., 1995	60 min	-0.848	5	0.159
		20	Layer et al., 1995	60 min	-1.191	5	0.059
		40	Layer et al., 1995	60 min	-0.928	5	0.127
		○	**Overall (4 studies)**	○	-0.894	○	0.040
	Ifenprodil	0.1	Ghasemi et al., 2010	45 min	-0.216	10	0.615
		1	Ghasemi et al., 2010	45 min	-1.394	10	0.004
		3	Ghasemi et al., 2010	45 min	-1.765	10	0.001
		10	Poleszak et al., 2013	60 min	-0.334	9	0.461
		0.5	Ghasemi et al., 2010	45 min	-0.737	10	0.097
		○	**Overall (5 studies)**	○	-0.864	○	0.013
	Traxoprodil	0.3	Serefko et al., 2016	60 min	0.335	9	0.460
		3	Bristow et al., 2017	15 min	-1.229	8	0.018
		5	Poleszak et al., 2016	60 min	0.256	8	0.589
		10	Stasiuk et al., 2017	60 min	0.000	10	1.000
			Poleszak et al., 2016	60 min	0.410	8	0.391
		20	Poleszak et al., 2016	60 min	-2.101	8	0.000
		40	Poleszak et al., 2016	60 min	-3.128	8	0.000
		○	**Overall (7 studies)**	○	-0.606	○	0.049
	Ro-25-6981	6	Zhang et al., 2013	75 min	-0.299	10	0.488
	L-701,324	1	Wlaz et al., 2011	60 min	-0.334	8	0.483
			Szewczyk et al., 2010	60 min	-0.256	8	0.589
			Serefko et al., 2016	60 min	0.000	9	1.000
			Poleszak et al., 2013	60 min	0.000	8	1.000
			Poleszak et al., 2011	60 min	-0.048	8	0.922
		4	Poleszak et al., 2007	60 min	-2.574	8	0.000
		○	**Overall (6 studies)**	○	-0.445	○	0.173
	2-MeO-NEK	2.5	Sayson et al., 2019	30 min	-1.964	10	0.000
		5	Sayson et al., 2019	30 min	-1.754	10	0.001
		10	Sayson et al., 2019	15 min	-1.413	8	0.008
					-2.855	8	0.000
				30 min	-3.573	10	0.000
		○	**Overall (5 studies)**	○	-2.213	○	0.000
	4-Cl-KYN	0.2	Zanos et al., 2015	60 min	-0.627	12	0.121
		1	Zanos et al., 2015	60 min	-0.669	12	0.099
		5	Zanos et al., 2015	60 min	-1.044	12	0.013
		25	Zanos et al., 2015	24 hours	-2.040	10	0.000
				60 min	-1.684	12	0.000
		125	Zanos et al., 2015	24 hours	-2.897	10	0.000
				60 min	-1.340	12	0.002
		○	**Overall (7 studies)**	○	-1.390	○	0.000
	4-MeO-NEK	2.5	Sayson et al., 2019	30 min	-1.749	10	0.001
		5	Sayson et al., 2019	30 min	-2.017	10	0.000
		10	Sayson et al., 2019	15 min	-1.749	8	0.002
					-2.560	8	0.000
				30 min	-3.399	10	0.000
		○	**Overall (5 studies)**	○	-2.234	○	0.000
	CGP-37849	0.3	Wolak et al., 2015	60 min	0.351	9	0.439
			Wolak et al., 2013	60 min	-0.227	10	0.597
			Poleszak et al., 2013	60 min	0.000	9	1.000
		0.625	Poleszak et al., 2007	60 min	-1.827	8	0.001
		0.635	Dybala et al., 2008	30 min	-2.755	6	0.000
		○	**Overall (5 studies)**	○	-0.683	○	0.061
	Magnesium Sulphate	10	Haj-Mirzaian et al., 2015	30 min	-0.309	6	0.564
		30	Haj-Mirzaian et al., 2015	30 min	-0.979	6	0.085
	NENK	2.5	Sayson et al., 2019	30 min	-1.453	10	0.003
		5	Sayson et al., 2019	30 min	-3.016	10	0.000
		10	Sayson et al., 2019	15 min	-1.477	8	0.006
					-1.938	8	0.001
				30 min	-2.110	10	0.000
		○	**Overall (5 studies)**	○	-1.959	○	0.000
	CPP	10	Zhang et al., 2013	105 min	-0.046	10	0.914
	MXE	2.5	Botanas et al., 2017	24 hours	-0.462	15	0.200
		5	Botanas et al., 2017	24 hours	-1.350	15	0.001
		10	Botanas et al., 2017	24 hours	-1.634	15	0.000
	NVP-AAM077	1.2	Zhang et al., 2013	90 min	-0.302	10	0.483
	(2S,6S)-HNK	20	Yokoyama et al., 2020	30 min	-0.248	12	0.532
	(2R,6R)-HNK	20	Yokoyama et al., 2020	30 min	-0.031	12	0.937
	(R)-NK	20	Yokoyama et al., 2020	30 min	-0.345	12	0.385
	(S)-NK	20	Yokoyama et al., 2020	30 min	-0.557	12	0.166
	DHNK	50	Salat et al., 2015	7 days	1.300	7	0.020
	Norketamine	5	Salat et al., 2015	30 min	0.122	8	0.797
		10	Salat et al., 2015	30 min	0.525	8	0.276
		50	Salat et al., 2015	30 min	-1.094	8	0.032
TST	Eliprodil	2.5	Layer et al., 1995	30 min	-0.177	5	0.757
		5	Layer et al., 1995	30 min	-1.100	5	0.077
		10	Layer et al., 1995	30 min	-0.571	5	0.330
		20	Layer et al., 1995	30 min	0.957	5	0.117
		○	**Overall (4 studies)**	○	-0.222	○	0.703
	Ro-25-6981	5	Skolnick et al., 2015	45 min	-2.027	8	0.001
		10	Skolnick et al., 2015	45 min	-3.297	8	0.000
	2-MeO-NEK	2.5	Sayson et al., 2019	30 min	-0.963	10	0.034
		5	Sayson et al., 2019	30 min	-1.825	10	0.000
		10	Sayson et al., 2019	30 min	-1.408	10	0.004
	4-Cl-KYN	5	Zanos et al., 2015	60 min	-1.482	16	0.000
		25	Zanos et al., 2015	60 min	-2.117	16	0.000
		125	Zanos et al., 2015	60 min	-1.909	16	0.000
			Yokoyama et al., 2020	60 min	-1.909	16	0.000
		○	**Overall (4 studies)**	○	-1.852	○	0.001
	4-MeO-NEK	2.5	Sayson et al., 2019	30 min	-1.347	10	0.005
		5	Sayson et al., 2019	30 min	-1.078	10	0.019
		10	Sayson et al., 2019	30 min	-1.114	10	0.016
	Acamprosate	25	Palucha-Poniewiera et al., 2012	60 min	-0.787	8	0.110
		50	Palucha-Poniewiera et al., 2012	60 min	-1.393	8	0.009
		100	Palucha-Poniewiera et al., 2012	60 min	-3.329	8	0.000
		200	Palucha-Poniewiera et al., 2012	60 min	-2.553	8	0.000
		400	Palucha-Poniewiera et al., 2012	60 min	-3.278	8	0.000
		○	**Overall (5 studies)**	○	-2.170	○	0.000
	Magnesium Sulphate	5	Haj-Mirzaian et al., 2015	30 min	-0.257	6	0.631
		15	Haj-Mirzaian et al., 2015	30 min	-1.081	6	0.061
	Memantine	2.5	Kos et al., 2005	40 min	-1.149	10	0.013
		5	Kos et al., 2005	40 min	-2.496	10	0.000
		7.5	Kos et al., 2005	40 min	-4.160	10	0.000
		10	Skolnick et al., 2015	45 min	-3.289	10	0.000
		15	Kos et al., 2005	40 min	-5.377	10	0.000
		20	Skolnick et al., 2015	45 min	-4.071	8	0.000
		1.8	Kos et al., 2005	40 min	-0.192	10	0.655
		3.7	Kos et al., 2005	40 min	-1.916	10	0.000
		○	**Overall (8 studies)**	○	-2.580	○	0.000
	NENK	2.5	Sayson et al., 2019	30 min	-0.586	10	0.181
		5	Sayson et al., 2019	30 min	-0.732	10	0.099
		10	Sayson et al., 2019	30 min	-1.029	10	0.025
	Lanicemine	18	Skolnick et al., 2015	45 min	-3.832	10	0.000
		4.5	Skolnick et al., 2015	45 min	-0.826	10	0.065
		9	Skolnick et al., 2015	45 min	-3.268	10	0.000
	Arcaine	1	Cunha et al., 2015 (B)	90 min	-0.218	8	0.645
	MXE	2.5	Botanas et al., 2017	24 hours	-0.131	15	0.713
		5	Botanas et al., 2017	24 hours	-1.350	15	0.001
		10	Botanas et al., 2017	24 hours	-1.413	15	0.000

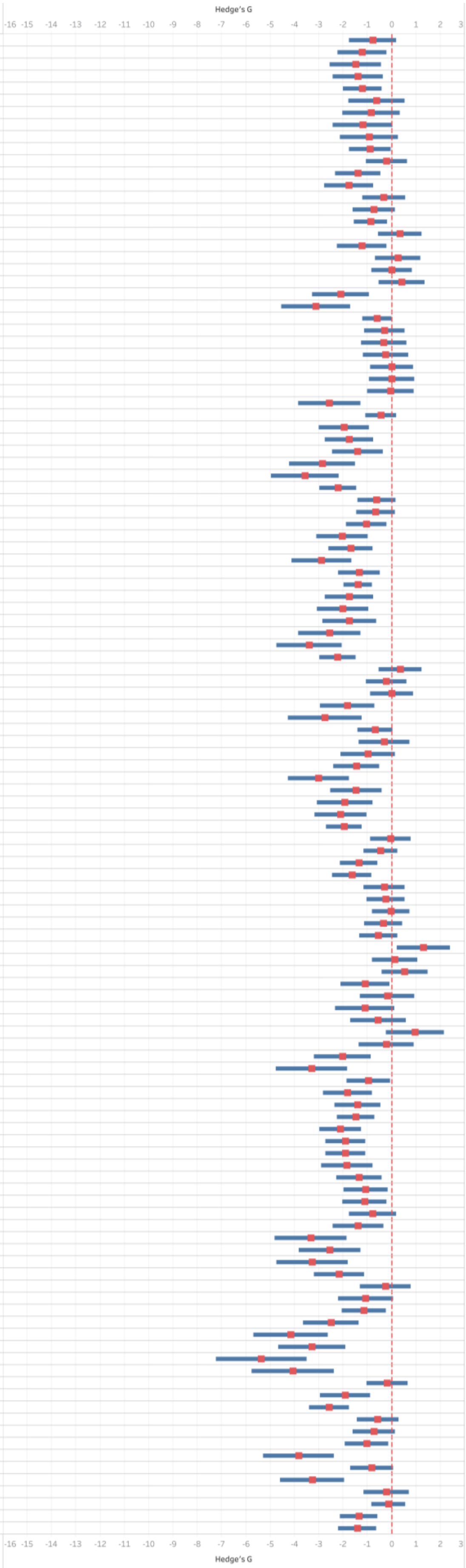

Supplementary figure 1.3 Forest plot showing effects of treatment with other NMDAR antagonists in the TST and FST subdivided by pharmacological agent, dose and pre-treatment time. Where each drug has been investigated in multiple studies these are shown as each individual study.

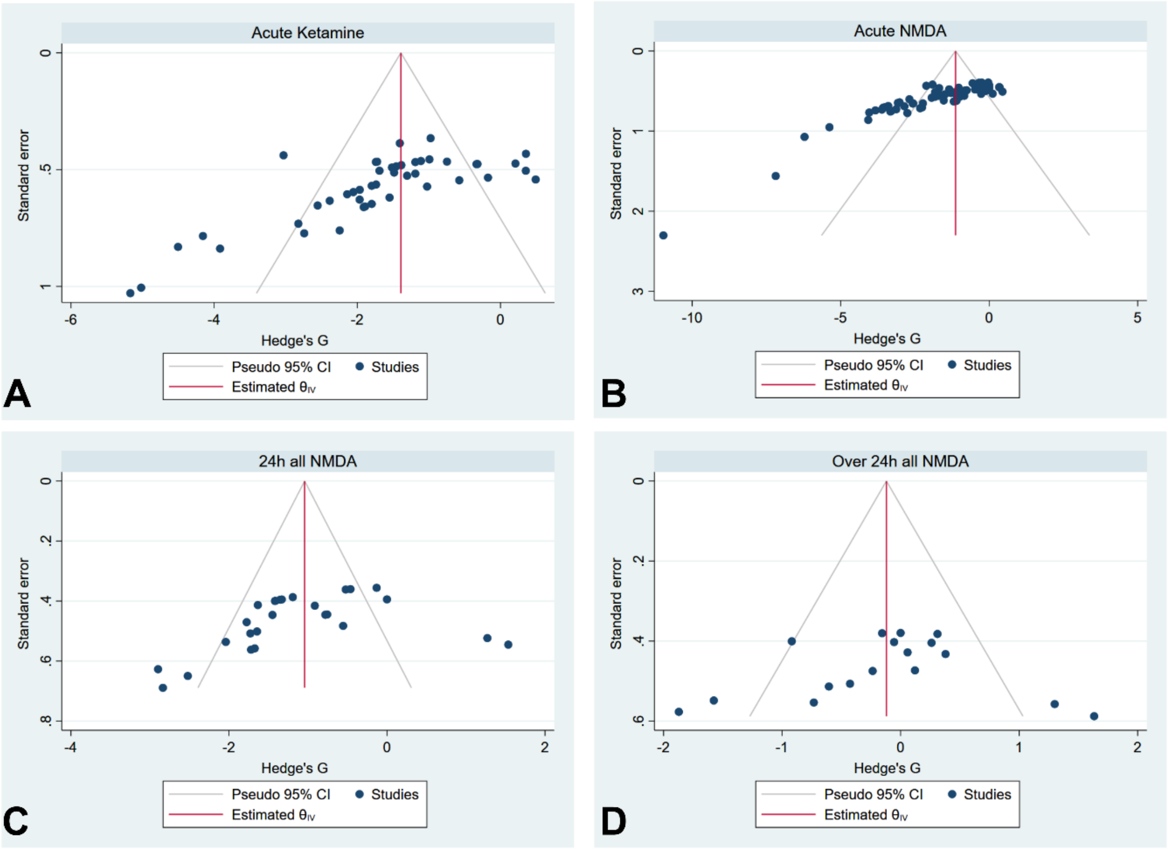

Supplementary figure 2.1 Funnel plots of studies investigating effects of ketamine, MK-801 and other NMDAR antagonists acutely, after 24 hours and after over 24 hours with Hedge's G plotted against the standard error. Entries are for **(A)** effective doses of ketamine between 0.1 and 30 mg/kg at acute pretreatment times (<60 min) **(B)** MK-801 + other NMDAR antagonists administered acutely, **(C)** all NMDAR antagonists at all doses at 24 hours and **(D)** over 24 hours.

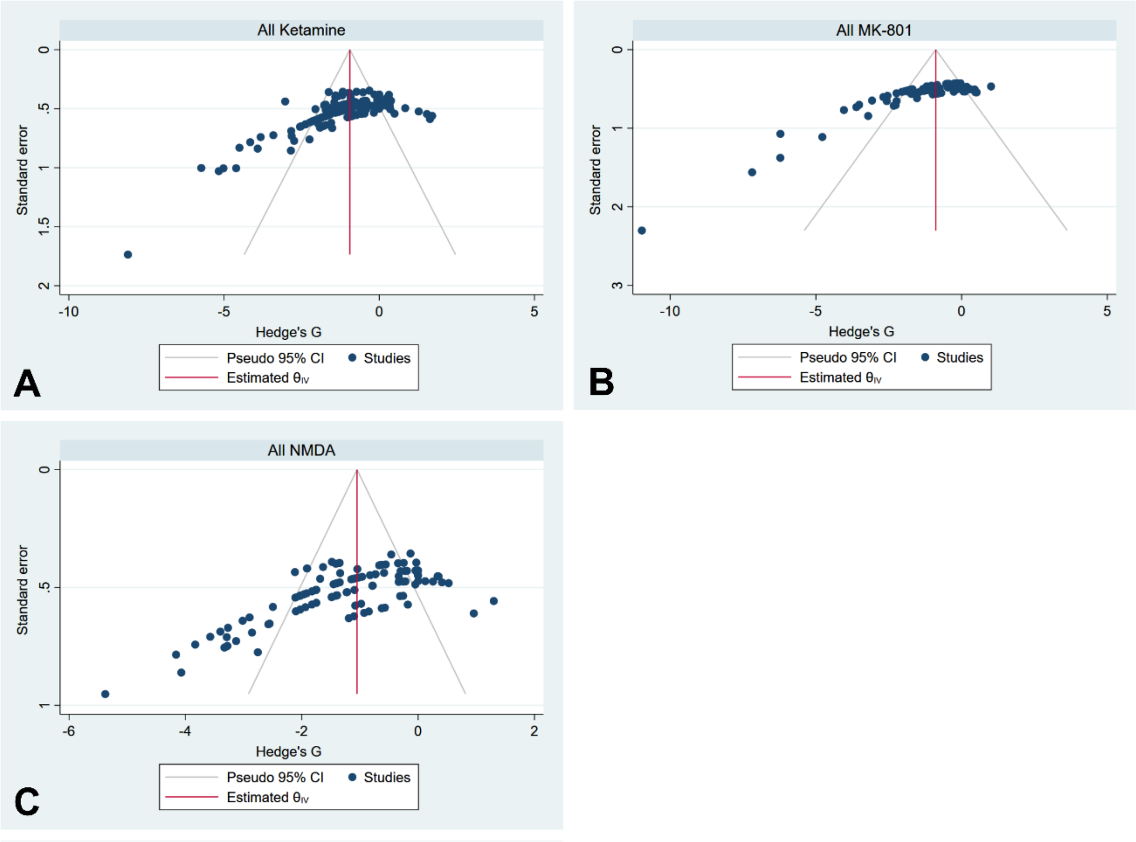

Supplementary figure 2.2 Funnel plots of all studies investigating effects of ketamine, MK-801 and other NMDAR antagonists. (A) all ketamine doses at all pretreatment times, **(B)** all MK-801 doses at all pretreatment times, **(C)** all other NMDAR antagonists at all doses and all pretreatment times, **(D)** anaesthetic doses of ketamine at all pretreatment times, **(E)** sub-effective doses of ketamine at all pretreatment times.

Drug	Pre-Treatment (hrs)	Heterogeneity			Publication bias	
		I-squared (%)	p value	df	Egger's intercept	p value
Ketamine	≤ 1	71.9	<0.0001	44	-5.67	<0.0001
MK-801	≤ 1	73.9	<0.0001	32	-7.99	<0.0001
Other NMDAR antagonists	≤ 1	80.6	<0.0001	43	-8.20	<0.0001
All NMDAR antagonists	24	73.5	<0.0001	25	-3.22	0.18
All NMDAR antagonists	> 24	76.0	<0.0001	16	-4.74	0.053
Anaesthetic ketamine	--	82.5	<0.0001	17	-8.69	0.001
Sub-clinical ketamine	--	46.4	0.083	6	-9.61	0.14
All ketamine	--	76.2	<0.0001	140	-5.80	<0.0001
All MK-801	--	77.8	<0.0001	73	-7.72	<0.0001
All other NMDAR antagonists	--	75.0	<0.0001	109	-6.72	<0.0001

Supplementary Table 2.3 Heterogeneity and publication bias estimates for each drug at different pre-treatment times.

Author	Year	Allocation sequence generated adequately?	Groups similar at baseline or adjusted for confounders?	Allocation adequately concealed?	Animals randomly housed during the experiment?	Investigators blinded?	Animals selected at random?	Outcome assessor blinded?	Incomplete outcome data adequately addressed?	Reports of the study free of selective outcome reporting?	Study free of other problems that could result in the high risk of bias?
Andreasen et al.	2013	NA	Yes	NA	NA	NA	NA	NA	Yes	Yes	Yes
Antony et al.	2014	Yes	Yes	Yes	NA	Yes	Yes	Yes	Yes	Yes	Yes
Bettio et al.	2012	NA	Yes	NA	NA	Yes	NA	Yes	Yes	Yes	Yes
Botanas et al.	2017	NA	Yes	NA	NA	Yes	NA	Yes	Yes	Yes	Yes
Bristow et al.	2017	Yes	Yes	Yes	NA	Yes	Yes	Yes	Yes	Yes	Yes
Cruz et al.	2009	Yes	Yes	Yes	NA	Yes	NA	Yes	Yes	Yes	No
Cunha et al. (1)	2015	Yes	Yes	NA	NA	Yes	Yes	Yes	Yes	Yes	Yes
Cunha et al. (2)	2015	Yes	Yes	Yes	NA	Yes	Yes	Yes	Yes	Yes	Yes
Dhir et al.	2008	NA	Yes	NA	NA	NA	NA	NA	NA	NA	NA
do Vale et al.	2016	NA	Yes	NA	NA	NA	NA	NA	Yes	Yes	Yes
Dybala et al.	2008	NA	Yes	NA	NA	NA	NA	NA	No	No	Yes
Ebrahimi-Ghiri et al.	2019	NA	Yes	NA	NA	NA	NA	Yes	Yes	Yes	Yes
Fitzgerald et al.	2019	NA	Yes	NA	NA	NA	NA	Yes	Yes	Yes	Yes
Fukumoto et al.	2016	NA	Yes	NA	NA	Yes	NA	Yes	No	Yes	Yes
Fukumoto et al.	2017	NA	Yes	NA	NA	Yes	NA	Yes	Yes	Yes	Yes
Ghasemi et al.	2010	NA	Yes	NA	NA	NA	NA	NA	Yes	Yes	Yes
Haj-Mirzaian et al.	2015	NA	Yes	NA	NA	NA	NA	NA	Yes	Yes	Yes
Highland et al.	2018	NA	Yes	Yes	NA	Yes	Yes	Yes	Yes	Yes	Yes
Holanda et al.	2018	Yes	Yes	NA	NA	NA	Yes	NA	Yes	Yes	Yes
Kaster et al.	2012	NA	Yes	NA	NA	NA	NA	NA	Yes	Yes	Yes
Khakpai et al.	2019	NA	Yes	NA	NA	NA	NA	NA	Yes	Yes	NA
Khan et al.	2016	NA	Yes	NA	NA	NA	NA	NA	Yes	Yes	Yes
Kos et al.	2005	NA	Yes	NA	NA	NA	NA	NA	Yes	Yes	Yes
Landrigan et al.	2018	NA	Yes	NA	NA	NA	NA	NA	Yes	Yes	No
Layer et al.	1995	NA	No	NA	NA	NA	NA	NA	Yes	Yes	Yes
Li et al.	2015	NA	Yes	NA	NA	Yes	NA	Yes	Yes	Yes	Yes
Li et al.	2019	NA	Yes	NA	NA	NA	NA	NA	Yes	Yes	Yes
Lin et al. (1)	2016	NA	Yes	NA	NA	NA	NA	NA	Yes	Yes	No
Lin et al. (2)	2016	Yes	Yes	Yes	NA	Yes	Yes	Yes	Yes	Yes	No
Ludka et al.	2016	NA	Yes	NA	NA	NA	NA	NA	Yes	Yes	Yes
Mantovani et al.	2003	NA	Yes	NA	NA	NA	NA	NA	Yes	No	Yes
Mohseni et al.	2017	NA	Yes	NA	NA	NA	NA	NA	Yes	Yes	Yes
Neis et al.	2015	NA	Yes	NA	NA	NA	NA	NA	Yes	Yes	Yes
Ostadhadi et al. (1)	2016	NA	Yes	NA	NA	NA	NA	NA	Yes	Yes	Yes
Ostadhadi et al. (2)	2016	NA	Yes	NA	NA	NA	NA	NA	Yes	Yes	Yes
Ostadhadi et al. (3)	2016	NA	Yes	NA	NA	NA	NA	NA	Yes	Yes	Yes
Ostadhadi et al.	2017	NA	Yes	NA	NA	NA	NA	NA	Yes	Yes	Yes
Palucha-Poniewiera	2012	NA	Yes	NA	NA	NA	NA	NA	Yes	Yes	Yes
Panconi et al.	1993	NA	Yes	NA	NA	Yes	NA	NA	Yes	Yes	Yes
Pochwat et al.	2018	NA	Yes	NA	NA	NA	NA	NA	Yes	Yes	Yes
Poleszak et al.	2007	NA	Yes	NA	NA	NA	NA	NA	Yes	Yes	Yes
Poleszak et al.	2011	NA	Yes	NA	NA	NA	NA	NA	Yes	Yes	Yes
Poleszak et al.	2013	Yes	Yes	NA	NA	NA	Yes	NA	Yes	Yes	Yes
Poleszak et al.	2016	NA	Yes	NA	NA	NA	NA	NA	Yes	Yes	Yes
Popik et al.	2008	NA	Yes	NA	NA	Yes	NA	Yes	Yes	Yes	Yes
Rieger et al.	2014	NA	Yes	NA	NA	NA	NA	NA	Yes	Yes	Yes
Robson et al.	2012	NA	Yes	NA	NA	NA	NA	NA	Yes	Yes	Yes
Rosa et al.	2003	NA	Yes	NA	NA	NA	NA	NA	Yes	Yes	Yes
Rosa et al.	2016	NA	Yes	NA	NA	Yes	NA	Yes	Yes	Yes	Yes
Sakhaee et al.	2017	NA	Yes	NA	NA	NA	NA	NA	Yes	Yes	Yes
Salat et al.	2015	NA	Yes	NA	NA	NA	NA	NA	Yes	Yes	Yes
Salehi-Sadaghiani et	2012	NA	Yes	NA	NA	NA	NA	NA	Yes	Yes	Yes
Sasaki-Hamada et al.	2019	NA	Yes	NA	NA	NA	NA	Yes	No	No	Yes
Sayson et al.	2019	NA	Yes	NA	NA	NA	NA	NA	Yes	Yes	Yes
Serefko et al.	2016	Yes	Yes	NA	Yes	NA	Yes	NA	Yes	Yes	Yes
Skolnick et al.	2015	NA	Yes	NA	NA	Yes	NA	Yes	Yes	Yes	Yes
Stasiuk et al.	2017	NA	Yes	NA	NA	NA	NA	NA	Yes	Yes	Yes
Szewczyk et al.	2010	NA	Yes	NA	NA	NA	NA	NA	Yes	Yes	Yes
Wilson et al.	2020	NA	Yes	NA	NA	NA	Yes	NA	Yes	Yes	Yes
Wlaz et al.	2011	NA	Yes	NA	NA	NA	NA	NA	Yes	Yes	Yes
Wolak et al.	2013	NA	Yes	NA	NA	NA	NA	NA	Yes	Yes	Yes
Wolak et al.	2015	NA	Yes	NA	NA	NA	NA	NA	Yes	Yes	Yes
Yokoyama et al.	2020	NA	Yes	NA	NA	NA	NA	Yes	Yes	Yes	Yes
Zanos et al.	2015	NA	NA	NA	NA	Yes	NA	Yes	Yes	Yes	Yes
Zanos et al.	2017	NA	NA	NA	NA	Yes	NA	Yes	Yes	Yes	No
Zhang et al.	2013	NA	Yes	NA	NA	Yes	NA	Yes	Yes	Yes	No

Supplementary table 3.1 List of included studies including risk of bias measures.
